# Regulation of Peptidase Activity beyond the Active Site in Human Health and Disease

**DOI:** 10.3390/ijms242317120

**Published:** 2023-12-04

**Authors:** Ana Obaha, Marko Novinec

**Affiliations:** Faculty of Chemistry and Chemical Technology, Department of Chemistry and Biochemistry, University of Ljubljana, Večna pot 113, SI-1000 Ljubljana, Slovenia; ana.obaha@fkkt.uni-lj.si

**Keywords:** protease, inhibition, allostery, exosite, glycosaminoglycan, interaction

## Abstract

This comprehensive review addresses the intricate and multifaceted regulation of peptidase activity in human health and disease, providing a comprehensive investigation that extends well beyond the boundaries of the active site. Our review focuses on multiple mechanisms and highlights the important role of exosites, allosteric sites, and processes involved in zymogen activation. These mechanisms play a central role in shaping the complex world of peptidase function and are promising potential targets for the development of innovative drugs and therapeutic interventions. The review also briefly discusses the influence of glycosaminoglycans and non-inhibitory binding proteins on enzyme activities. Understanding their role may be a crucial factor in the development of therapeutic strategies. By elucidating the intricate web of regulatory mechanisms that control peptidase activity, this review deepens our understanding in this field and provides a roadmap for various strategies to influence and modulate peptidase activity.

## 1. Introduction

Peptidases, enzymes that cleave the peptide bond of (poly)peptides, are ubiquitous in nature. They are found in all three domains of life as well as viruses, and they make up a significant proportion of the enzymes found in living organisms. The MEROPS database (available online at https://www.ebi.ac.uk/merops/, accessed on 7 March 2023) catalogues over 4000 peptidases, of which approximately 700 are found in humans [1]. This wide range comprises peptidases of seven different catalytic types, i.e., serine, cysteine, aspartic, glutamic, asparagine and metallopeptidases, underscoring the multifaceted role that these enzymes play in regulating important physiological processes such as blood coagulation and apoptosis as well as nonspecific protein turnover. Given their diverse roles, a tight regulation of peptidase activity is required, and their dysregulation has been linked to the development or progression of numerous diseases, including autoimmune diseases, cancer, cardiovascular disease, and bacterial or viral infections. As of 2022, 25 drugs targeting peptidases were in clinical use and nearly 100 others were in various stages of clinical testing [2].

**Figure 1:**
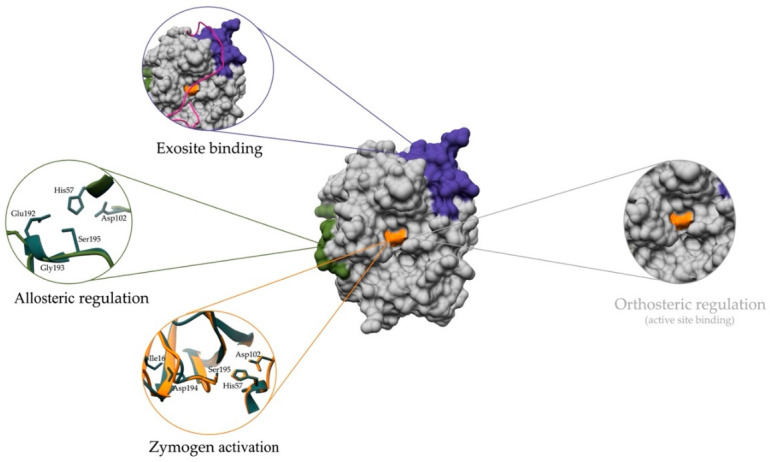
Mechanisms of regulation of proteolytic enzymes illustrated using the example of human thrombin. Orthosteric regulation, in which an effector is bound to the active site, is an established approach to modulate enzyme activity. However, our understanding goes beyond the conventional paradigm and also includes complicated mechanisms that go beyond the active site. In allosteric regulation, enzyme activity is modulated by binding to a site other than the active site, which changes the conformation of the enzyme. Regulation by exosites involves interactions with specific regions outside the active site that influence the activity and function of the enzyme. Zymogen activation refers to the process by which a peptidase precursor is converted to its active form through self-cleavage or other activation mechanisms. Taken together, these different regulatory mechanisms influence the activity and specificity of the enzyme.

The regulation of proteolytic enzymes occurs at several levels, starting with transcription. Many proteolytic enzymes are expressed as inactive zymogens that require specific conditions for activation. After activation, peptidase activity can be modulated by ligands that bind to the active site or other areas of the enzyme surface, such as exosites (i.e., additional substrate binding sites distant from the active site) and allosteric regulatory sites. Several mechanisms can be involved in the regulation of one and the same peptidase, as illustrated on the example of thrombin in Figure 1 [3]. The modulators can be organic molecules, peptides, or entire proteins of endogenous or synthetic origin, and they can influence not only the enzyme activity but also the specificity of the peptidase. The vast majority of modulators targeting peptidases for medicinal and/or research purposes are inhibitors that bind to the active site in substrate-like manner, thereby abolishing enzyme activity. While this is a proven and effective strategy in some cases, like the gliptin class of dipeptidyl-peptidase 4 inhibitors for the treatment of type 2 diabetes [4], it can also cause significant on or off-target side effects that can ultimately prohibit the use of the inhibitor in the clinic. For example, the cathepsin K inhibitor odanacatib (Merck & Co., Rahway, NJ, USA) was terminated after completing phase 3 clinical trials for the treatment of post-menopausal osteoporosis due to rare but significant side effects, despite continued demonstration of its efficacy [5]. Exploring alternative ways of regulating peptidase activity in such cases could prove beneficial in comparison to active site-directed inhibitors.

The aim of this review is to evaluate a range of peptidase–modifier interactions by examining and comparing some recently discovered and common mechanisms, such as the activation of zymogens, binding of glycosaminoglycans, regulation via exosites and allosteric sites, and non-inhibitory binding proteins. We concentrate on interactions relevant to human physiology and pathology in order to provide an expert opinion on whether these strategies can be used to regulate peptidase activity in vivo.

## 2. Regulating the Activation and Maturation of Zymogens

One of the most fundamental and widespread mechanisms for protection against excessive proteolysis is the synthesis and secretion of peptidases in the form of inactive precursors called zymogens. These inactive peptidases are stored until activation signals are received, allowing for rapid recruitment without the need for prior transcription. Zymogens contain inhibitory (pro)peptides that prevent substrate binding and proteolytic activity. Therefore, the inhibition of activation is a viable strategy for preventing peptidase activity in general.

**Figure 2:**
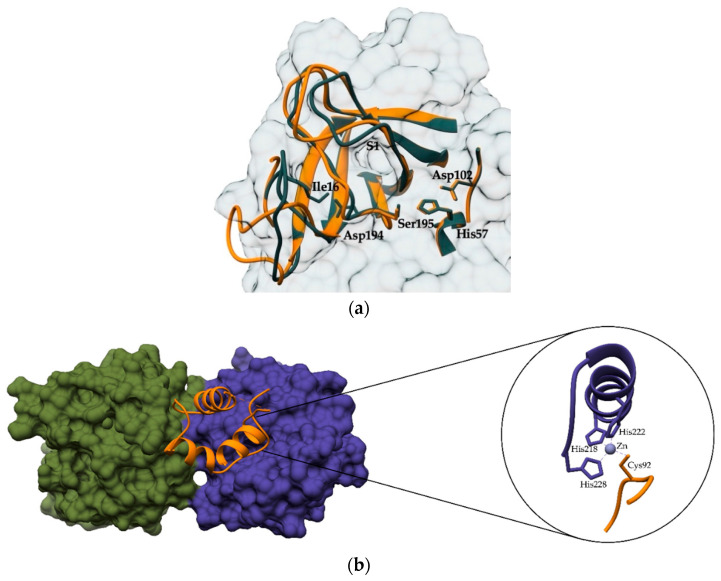
Crystal structures of zymogens. (**a**) Superposition of the active sites of the serine peptidase trypsinogen (orange; PDB 1TGN) and trypsin (gray; PDB 1MCT). Residues His57, Asp102, and Ser195 form the active site. After cleavage of the activation hexapeptide, the newly formed N-terminus (Ile16) forms a salt bridge with Asp194, leading to a conformational change in the S1 binding pocket. (**b**) Structure of MMP-1 (PDB 1SU3), a collagenolytic member of the matrix metalloprotease family. It is a multidomain protein consisting of a propeptide (orange), a hemopexin domain (green), and a catalytic domain (blue). The free Cys92 residue in the propeptide (orange) of MMP-1 interacts as a fourth ligand for the zinc ion in the active site. The cysteine switch excludes water from the coordination sphere of zinc inactivating metalloproteinase.

One of the earliest studies of zymogen activation, which incidentally is quite different from other peptidase families, is the chymotrypsin-like serine peptidases. Here, zymogen inactivity results from incomplete folding of the peptidase (Figure 2a) rather than physical closure of the active site by the proregion. Importantly, the S1 substrate-binding site and the oxyanion hole are not formed in the inactive state. Once the N-terminal hexapeptide, known as the activation peptide, is cleaved, the newly formed N-terminal residue forms a salt bridge that triggers a conformational change leading to the formation of the active site [6,7,8]. The activation peptide can be cleaved by autocatalysis or by other peptidases [9,10,11,12]. An example of clinically relevant activators of chymtrypsin-like serine peptidases is dipeptidyl peptidase I (DPPI, also known as cathepsin C), which activates most of the immune system serine peptidases, including granzymes [13], neutrophil serine peptidases [14], and mast cell chymase [15]. As an important catalyst for the activation of peptidases in immune cells, DPPI is a promising target for the treatment of inflammatory diseases characterized by immune system overactivity. To date, numerous DPPI inhibitors have been developed and tested (see [16] for a recent review). The most advanced candidate for clinical use, brensocatib (Insmed, Inc., Bridgewater, NJ, USA), is currently being tested in a phase 3 clinical trial for the treatment of non-cystic fibrosis bronchiectasis, following the successful completion of a phase 2 trial [17].

DPPI belongs to the papain-like peptidase family, which includes members collectively known as cysteine cathepsins in animals. Most cysteine cathepsins (but not DPPI) are capable of autocatalytic activation. Their proregion is reversibly linked to the fully formed active site and physically blocks the cleft, preventing substrate binding. The activation mechanism of cysteine cathepsins has been extensively studied in procathepsin B, which is a cysteine peptidase involved in intracellular catabolism, the regulation of angiogenesis [18], neurogenesis [19], and also tumor growth and invasion [20,21]. Binding of the proregion is pH dependent [22], and at acidic pH, the electrostatic interactions between the residues in the proregion and the catalytic cleft are disrupted, leading to the release of the proregion and the formation of an intermediate structure, the active zymogen. This active zymogen can then activate adjacent proenzyme molecules by proteolytic cleavage of their proregion [23] in a bimolecular process [24]. Cathepsin K is another well-studied cysteine cathepsin involved in bone remodeling that is capable of autocatalytic processing in acidic bone remodeling lacunae [25]. Other lysosomal cysteine cathepsins such as L [26,27,28], S [29] and F [30] are also capable of autocatalytic activation. 

Some cysteine cathepsins cannot undergo autocatalytic processing, such as human cathepsin X, which has a short proregion covalently linked to the active site via an unbound cysteine residue in the proregion and a cysteine residue in the catalytic diad [31]. As a pure exopeptidase, cathepsin X is processed by other cysteine peptidases, such as cathepsin L [32]. Similarly, DPPI cannot be autocatalytically activated. DPPI has a unique additional exclusion domain that converts its typical papain-like endopeptidase activity to a dipeptidylpeptidase [33,34], and it is activated by either cathepsin L or S [35]. Procathepsin H is another papain-like cysteine peptidase that stands out from the family, as it is cleaved at three different sites upon activation. The resulting active enzyme contains both a catalytically active domain and a minichain derived from the propeptide that remains covalently bound to the enzyme. This minichain occludes the active site and enables the aminopeptidase activity of the peptidase [36]. Cathepsin L is believed to activate procathepsin H [37]. 

Similar binding of the unpaired cysteine residue as in cathepsin X can be observed in zymogens of matrix metalloproteases (MMPs; Figure 2b), where this highly conserved residue interacts as a fourth ligand for the zinc ion in the active site. This binding, termed the cysteine switch [38], excludes water from the coordination sphere of zinc (Figure 2b). To activate MMPs, this bond must be disrupted, and the thiol group of the cysteine must be replaced by a water molecule. This is accomplished by stepwise proteolytic cleavage followed by conformational changes [39]. Peptidases such as kallikreins and the MMPs themselves are involved in cleavage [40]. Cytotoxic oxidants such as hypochlorous acid, a product of myeloperoxidase, can also activate the cysteine switch in metalloproteases [41,42,43]. 

Aspartic peptidases, including pepsinogen and related peptidases, follow a similar activation process as cysteine peptidases. The proregion of pepsinogen is bound to the catalytic cleft by reversible electrostatic interactions [44]. Lowering the pH weakens these interactions and causes a conformational change [45]. This structural change creates a transition state that leads to the formation of the active enzyme through further proteolytic cleavage. Cleavage can occur either by autoproteolysis, as in the case of pepsinogen [46], or by other peptidases, as in the case of prorenin [47]. In the case of procathepsin D, which is also an aspartic peptidase, both autocatalytic processing and processing by other peptidases are possible [48,49,50,51]. However, the autocatalytic processing of procathepsin D requires the presence of sulfated glycosaminoglycans (GAGs) [52]. 

**Figure 3:**
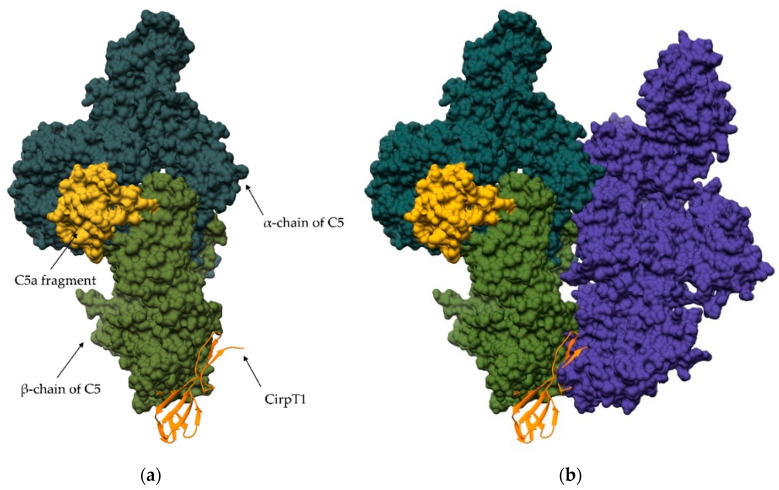
Inhibition of C5 zymogen activation by CirpT. The final stages of the complement activation pathway involve the activation of C5 by the peptidase C5 convertase. The convertase cleaves C5 into the proinflammatory fragments C5a and C5b. However, certain blood-sucking organisms have evolved a strategic mechanism to inhibit the activation of C5, effectively dampening the host immune response. One such inhibitor is CirpT1, which is secreted by the tick *Rhipicephalus puchellus*. (**a**) Binding of CirpT1 inhibitor (orange) to complement component C5 (PDB 6RQJ). Binding occurs via the β-chain of C5 (green). (**b**) Interaction between C5 and cobra venom factor (CVP; blue), which serves as an analog of the C3b fragment of C5 convertase (PDB 3PVM). The CVP or C5 convertase binding site coincides with the binding site of CirpT. This convergence of binding sites allows CirpT1 to effectively interfere with C5 activation [57].

Targeting zymogen activation may be a promising therapeutic strategy, but it is not always effective. For example, analyses of the inhibitory capacity and specificity of the proregion of individual members of the human cysteine cathepsins [53,54,55,56] have shown that for closely related family members such as cathepsins K, S, and L, selectivity is insufficient for specific protein targeting [56]. Targeting the activation of short-lived peptidases that would otherwise require high local concentrations of the inhibitor is likely to be more successful. Such peptidases are peptidases of the complement system, which is an important component of innate immunity. This strategy is employed in nature by tick *Rhipicephalus Pulchellus*, which has evolved a mechanism to inhibit host complement peptidases to evade the immune system. The saliva of the tick contains CirpT: short peptides that bind to the complement peptidase zymogen and prevent its activation (Figure 3) [57]. A similar effect is achieved with a small molecule that inhibits the activation of the matriptase zymogen [58]. Matriptase is a transmembrane serine peptidase involved in extracellular matrix degradation and epithelial migration and consequently involved in processes of cancer invasion and metastasis [59,60]. Inhibition of its activation is therefore a potentially clinically important process in the treatment of cancer. 

A potentially clinically useful regulator of zymogen activation is also a monoclonal antibody that binds to the proenzyme of urokinase-type plasminogen activator (uPA), which is an enzyme that converts plasminogen to plasmin [61] and is involved in tumor progression and metastasis. Binding of the antibody delays cleavage of the proregion and the subsequent activation of uPA [62]. Recently, a small molecule (JNJ0966) was found to inhibit the activation of metalloproteinase-9 (MMP-9), which is involved in several cancer processes [63,64,65]. This molecule binds near the cleavage site of the MMP-9 zymogen and represents a new pharmacological approach to inhibit this enzyme [66].

In certain cases, however, the goal is just the opposite, namely the enhanced activation of peptidase zymogens. A good example is the family of procaspases, which are cysteine peptidases and key components of apoptosis. Like most peptidases, caspases are expressed in the form of a zymogen. However, their activation process differs from that of the cysteine peptidases mentioned above. There are two subfamilies of caspases involved in the process of cell death: initiator and effector caspases. The initiator caspases, such as procaspase-9 and procaspase-8, are present in the cell in a zymogenic form and undergo autocatalytic cleavage upon receipt of a proapoptotic signal. In addition to autocatalytic cleavage of the zymogen, initiator caspases must form higher complexes with other proteins, such as the apoptosome, to achieve maximal activity [67,68]. Effector caspases, such as procaspase-3, are present in the cell in dimeric zymogen form [69] and are activated by initiator caspases [70]. The initiator caspase cleaves the executor procaspase, allowing the rearrangement of essential loops in the active site. This conformational change leads to the complete formation of the active site and subsequent activation of the peptidase [71]. The activation of effector caspases leads to the proteolytic cleavage of various substrates, resulting in cell death. In the treatment of cancer, inducing the death of cancer cells is desired. Therefore, drugs that stimulate the activation of effector procaspases are being sought. One such molecule is PAC-1, which activates procaspase-3 by forming a tight complex with an inhibitory zinc ion that allows procaspase-3 to activate itself into caspase-3 [72,73]. Another molecule that stimulates procaspase-3 activation is 1541B, which promotes fibril formation and the colocalization of effector and initiator caspases [74,75,76]. These molecules could be used as co-therapies for cancer treatment because they synergistically enhance procaspase-3 activation [77]. 

Finally, it should be noted that while most peptidases are synthesized as zymogens, there are exceptions to this rule. One such example that is also present in humans is the C1B family, which includes the eukaryotic bleomycin hydrolase [78] that is expressed without a proregion. In addition, viral peptidases are often expressed without a proregion—usually as part of polyprotein chains. This phenomenon can be observed, for example, in herpes simplex virus peptidase 1 (HSV-1) [79], human immunodeficiency virus peptidase 1 (HIV-1) [80], human cytomegalovirus peptidase (hCMV) [81], and the main protease (Mpro) of SARS-CoV-2 [82]. These peptidases become catalytically active after homodimerization or interaction with other modulators. Therefore, a promising strategy for antiviral drugs would be to prevent dimerization of the peptidase (see reviews [83,84]). This would not only solve the problem of antiviral drug resistance but also the problem of lack of specificity of orthosteric drugs. A few studies to prevent dimerization have been performed on HIV-1 and Mpro [85,86,87], but to our knowledge, no such drug is currently in use. 

## 3. Regulation of Peptidase Activity by Glycosaminoglycans

After zymogen activation, peptidase activity can be affected by the presence of macromolecules in its environment, such as glycosaminoglycans (GAGs). GAGs are linear, negatively charged polysaccharides that are ubiquitous components of the extracellular matrix (ECM) as well as internal cellular compartments. They are distinguished from one another by the nature of repeating disaccharide units, degree of sulfation, position of sulfate groups, and resulting negative charge density. In mammals, there are six GAGs, namely chondroitin sulfate (CS), dermatan sulfate (DS), heparin (HP), heparan sulfate (HS), hyaluronan (HA), and keratan sulfate (KS). All are sulfated to some degree and bind to proteins to form proteoglycans, with the exception of HA, which is unsulfated and mostly in a free form [88]. GAGs exert their effects through interactions with various protein targets, including peptidases and their inhibitors. 

**Figure 4:**
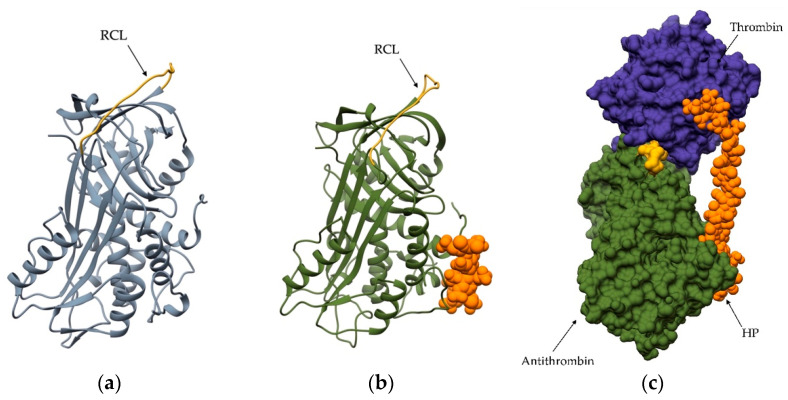
Thrombin inhibition by the antithrombin–heparin complex. (**a**) Structure of human antithrombin in its inactive state (PDB 1E05). (**b**) Structure of active human antithrombin in complex with a pentasaccharide (orange), which resembles the binding of HP (PDB 1E03). This binding leads to conformational changes, including changes in the reactive center loop (RCL; yellow), which is critical for thrombin recognition and inhibition. The HP-induced conformational change increases the inhibitory effect of antithrombin. (**c**) Complex of activated antithrombin (green), HP (orange), and thrombin (blue). RCL (yellow) represents a larger portion of the interaction surface between both proteins.

The most important GAG in the clinic is HP, which has been used for decades as an antithrombotic agent because it accelerates inactivation of the coagulation cascade. The latter involves the sequential activation of serine peptidases, with the final steps of the cascade consisting of the activation of prothrombin, which leads to active thrombin that cleaves fibrinogen to fibrin, the main component of a blood clot. The cascade of the blood-clotting process must be tightly regulated because excessive or insufficient activity of blood clotting factors can lead to thrombosis or hemophilia, respectively. One of the best-studied regulatory mechanisms is the effect of HP on antithrombin, which is a plasma glycoprotein that inhibits the activity of peptidases by forming an equimolar complex with the enzyme [89,90]. When antithrombin binds to HP, it undergoes a conformational change [91] that activates the protein and makes it a better thrombin inhibitor by increasing the accessibility of the inhibitor’s reactive bond to the enzyme (Figure 4). Binding to HP also makes antithrombin a better inhibitor of factor X [92], kallikrein [93,94], plasmin [95], and factor VII [96]. However, the effect of HP on the former factors is less pronounced than its effect on thrombin. 

Contrasting effects of GAGs have been demonstrated for serine peptidases stored in the secretory granules of mast cells and neutrophils, including chymase, tryptase, cathepsin G and elastase. The binding of HP to mast cell tryptase is critical for tetramerization and activation of the enzyme [97,98]. When the HP chains are too short to allow tetramerization, they enable the formation of an active monomer [99]. In addition, HP prevents the inhibition of mast cell chymase by its physiological inhibitors (α1-antitrypsin, α2-macroglobulin, and α2-antichymotrypsin) [100] but increases its susceptibility to inhibition by the secretory leukocyte proteinase inhibitor [101]. Similarly, the presence of HP decreases the inhibition of cathepsin G by α1-antichymotrypsin by sterically hindering the binding of the inhibitor [102], but it stimulates its inhibition by the mucus proteinase inhibitor (MPI) [103]. Similar effects have been observed for neutrophil elastase [104,105,106]. Moreover, HP directly inhibits the proteolytic activity of both cathepsin G and neutrophil elastase [107,108,109]. Neutrophil elastase in particular has been studied in detail with regard to its interactions with GAGs. It was found to be inhibited not only by HP but also by other sulfated GAGs. Kinetic experiments have shown that the mechanism of inhibition is typically a hyperbolic mixed inhibition with DS exhibiting the greatest inhibitory effect [110,111,112,113]. Interestingly, GAGs showed a biphasic effect on neutrophil elastase activity; i.e., they acted as inhibitors at low concentrations, but reactivation of the enzyme occurred at higher concentrations, which was attributed to multiple possible binding modes of GAGs to neutrophil elastase [112].

The interactions between GAGs and target proteins are electrostatic, mostly specific and depend on the degree and pattern of sulfation, resulting in different GAGs having different effects on the same target protein [88]. Cathepsin K is a good example of this phenomenon. In particular, chondroitin-4-sulfate (C4S) has the strongest stabilizing effect on this peptidase and increases its collagenolytic activity [114]. The presence of C4S has also been shown to be a necessary prerequisite for the collagenolytic activity of the enzyme, as the main function of the C4S/cathepsin K complex is to partially unfold the triple helix structure of collagen [115,116] by multimeric cathepsin K complexes formed in the presence of C4S [117,118]. Chondroitin-6-sulfate (C6S), which differs from C4S only by the position of the sulfate group at C6 instead of C4, has a 10-fold lower stabilizing effect on cathepsin K [119], demonstrating the specificity of GAGs for their target proteins. GAGs bind to cathepsin K at multiple sites away from the active site and affect enzyme activity by allosteric mechanisms [117,118,120]. The combination of experimental and computational data showed that cathepsin K switches between multiple conformational states and the binding of GAGs stabilizes its more active conformation [120]. In terms of enzyme kinetics, this manifests as the activation (increased activity) of cathepsin K on synthetic substrates [120]. However, differences were observed between the different types of GAGs at neutral pH, as CS and DS acted only as activators, while HP showed signs of diminishing activation effect and even inhibition at high concentrations under the same conditions, reflecting its multiple modes of interaction with cathepsin K [120].

It Is also interesting to note that GAGs have differential effects on closely related papain-like enzymes. While C4S activates cathepsin K, as discussed above, it has an inhibitory effect on the degradation of collagen IV by the closely related lysosomal cathepsin S [121]. Moreover, GAGs can affect the activity of inhibitors of papain-like peptidases but also their inhibitors with contrasting outcomes, similar as discussed above for serine peptidases of immune cells. For example, lysosomal cathepsin L, which is closely related to cathepsins K and S but is not directly regulated by GAGs, is indirectly affected by their interaction with cystatin C, which is a basic low molecular weight protein that acts as a general, emergency inhibitor of papain-like peptidases. In the presence of HP and heparan sulfate (HS), cystatin C cannot bind to the enzyme [122]. In contrast, HP enhances the inhibition of cathepsin L by serpins [123]. 

As mentioned earlier in this review, GAGs can affect zymogen activation. Studies have demonstrated that GAGs can promote the self-processing of several proenzymes such as procathepsin K [124], procathepsin B [125], and procathepsin S [126]. GAGs also accelerate the activation of certain metalloproteases (e.g., ADAMTS-5, ADAMTS-4, MMP-1, and MMP-2) [127,128,129,130,131] and aspartic peptidases (e.g., cathepsin D) [52]. 

In a summary of previous research, we have highlighted the complex and diverse effects of GAGs on proteolytic enzymes and emphasized the important role of these molecules in regulating proteolytic activity in both pathological and physiological processes. Therefore, the binding of GAGs to target peptidases represents a potential mechanism for regulating proteolytic activity, but their major limitation is their lack of specificity. Another consideration is that GAGs, as in the case of HP and cathepsin K, can bind to enzymes in different ways, resulting different effects on enzyme activity. 

## 4. Regulation via Exosites

**Figure 5:**
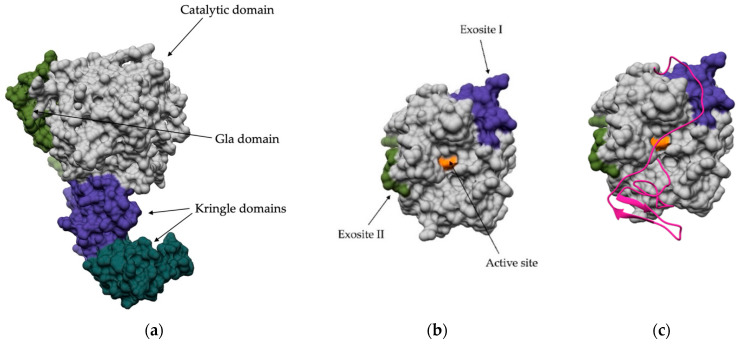
Crystal structure of thrombin. (**a**) Thrombin consists of four domains: the Gla domain, two kringle domains, and the trypsin-like catalytic domain. (**b**) Catalytic domain of thrombin, which includes two exosites in addition to the active site. Exosite I is located near the active site. Exosite II is located near the C-terminus. (**c**) Crystal structure of thrombin with bound huridin (magenta). Huridin is a natural inhibitor of thrombin isolated from the leech *Hirudo meidicinalis*. Huridin binds to the active site and exosite I of thrombin.

Exosites refer to specific sites of an enzyme that are outside the active site but still play an important role in binding substrates, cofactors, or other ligands. It is important to distinguish exosites from allosteric sites because exosites must normally be occupied for the enzyme to be active. In contrast, binding to the allosteric site only regulates the enzyme’s activity. The significance of exosites in peptidase activity has been studied mainly in the context of the blood coagulation cascade, where all the enzymes involved are trypsin-like serine peptidases. Despite their homology, they exhibit high specificity for biological substrates, which is determined not only by active site residues but also by exosites contributing to substrate affinity. The most important example for analysis of the influence of exosites on proteolytic activity is thrombin, which is the link between the intrinsic and extrinsic pathways of the coagulation cascade. The structure of prothrombin consists of four domains: the Gla domain, two kringle domains involved in protein–protein interactions, and the trypsin-like peptidase domain (Figure 5a). After initiation of the cascade, activated factor Xa cleaves two peptide bonds in prothrombin (Arg320-Ile321 and Arg271-Thr272) and generates active thrombin, which consists of a light and a heavy chain linked by a disulfide bond [132]. Activated thrombin has two distinct exosites that contribute to specific substrate binding and are both positively charged. Exosite I is adjacent to the active site cleft and binds various substrates, cofactors, and inhibitors such as fibrinogen [133], fibrin [134], heparin cofactor II [135], factor XI [136], and thrombomodulin [137]. The second site, referred to as exosite II, which is not an exosite site in the strict sense, as it is more of an allosteric site, is located near the C-terminal helix of the heavy chain. It has been identified as a binding site for HP [138,139] but is now known to bind other regulators such as glycoprotein Ibα [140] (Figure 5b).

Certain natural proteins found in the saliva of bloodsucking and venomous animals also bind to the exosites of thrombin and act as inhibitors or activators of blood coagulation. The first identified peptide of this type is the C-terminal portion of huridin, which is derived from the leech *Hirudo meidicinalis* and interacts with exosite I (Figure 5c). Although it does not completely inactivate thrombin, the peptide significantly reduces the binding of macromolecular substrates such as fibrinogen, while the peptidase continues to cleave synthetic peptides [141,142]. Bothrojaracin, isolated from the venomous snake *Bothops jararaca*, achieves a similar effect by binding to both exosites [143,144]. 

Several other serine peptidases of blood coagulation cascade, such as activated factor X [145], factor IX [146], and factor VII complexed with tissue factor [147], have also identified exosites. The exosite of factor X is also a target of the anticoagulant factor ixolaris, which is derived from the saliva of the tick *Ixodes scapularis*. Ixolaris, similar to tissue factor pathway inhibitor, binds to the exosite of factor X and inhibits activation by factor VII/tissue factor complex [148]. 

The presence of exosites has also been demonstrated in cysteine peptidases, including the elastolytic papain-like peptidase cathepsin V, which contains two exosites involved in elastin binding. The mutagenesis of these sites has been shown to result in a loss of elastolytic activity [149], highlighting the importance of exosites in determining enzyme specificity. Similarly, two exosites have been identified in the structure of cathepsin K, both of which are required for effective elastin degradation, whereas only one site is required for the collagenolytic activity of the enzyme. Importantly, the exosites in both cases are not involved in the cleavage of nonstructural substrates [150], making them a promising target for the development of inhibitors that would not interfere with the normal function of the enzyme. An example of such exosite-binding inhibitors is the tanshinones, a group of small plant molecules that selectively inhibit the collagenolytic activity of cathepsin K, demonstrating the potential therapeutic utility of targeting exosites in enzyme inhibition [151,152]. 

The presence of exosites has also been demonstrated for metalloproteases [153,154,155,156,157,158,159] involved in ECM remodeling and the activation of various biological molecules such as growth factors, cytokines, and factors involved in blood coagulation. Due to their multiple functions, they are involved in the development of various diseases, such as osteoarthritis, thrombosis, bleeding disorders, and cancer (for detailed information on the function of metalloproteases and pathologies, see references [160,161,162,163,164]). Therefore, they represent a promising target for the treatment of these diseases, although specificity remains a major challenge. The development of small molecules and antibodies targeting metalloproteases has therefore focused on binding to exosites, which are the main determinant of the specificity of these proteolytic enzymes. 

Researchers have succeeded in producing an antibody that specifically binds to the exosite of ADAMTS5 and inhibits its aggrecanase activity [165], making it a potential treatment option for osteoarthritis. The same peptidase can also be inhibited by glycoconjugated arylsulfonamines [166] and RNA aptamers [167]. Another metalloprotease involved in the development of osteoarthritis is MMP13 [168], whose activity has been successfully inhibited by small molecules with exosite binding [169,170,171,172,173]. Inhibitors in the form of antibodies have also been discovered that selectively inhibit the activity of MMP14, which is involved in the process of cell migration and invasion and whose excessive activity has been linked to the malignancy of various cancers [158,174,175,176]. In addition, specific short peptides have been synthesized that successfully inhibit the activity of MMP2 [177]. These examples suggest that targeting exosites of metalloproteases may be of clinical importance. A more detailed analysis of the inhibition of metalloproteases, including inhibition by exosite binding, can be found in references [178,179,180]. 

Targeting exosites is also a potential treatment for several infectious diseases, with one of the best-studied examples being *Clostridium botulinum* infection. The bacterium secretes botulinum neurotoxin serotype A, a zinc-dependent peptidase that specifically cleaves SNAP25 proteins in motor neurons, preventing the release of acetylcholine and leading to paralysis [181]. Currently, there are no drugs that inhibit the activity of the toxin already present in the neuron. In the search for inhibitors, the active site containing a zinc ion must be avoided because this metal is also present in human metalloproteases. For this reason, two specific exosites of the peptidase have shown great potential for inhibition [182,183], and several small molecules [184,185] and antibodies [186,187] have already been discovered that bind to either of the two. Similarly, the lethal metalloprotease of Bacillus anthracis has been inhibited by targeting its exosite [188]. 

## 5. Allosteric Regulation by Small Molecule Effectors

Allostery refers to the regulation of enzyme activity involving multiple spatially distant sites that influence each other via conformational changes. Originally, allostery was thought to occur only in oligomeric proteins, and models such as the Monod–Wyman–Changeux model [189] and the Koshland–Nemethy–Filmer model [190] were proposed to describe the relationships between enzyme activity and conformational changes. However, it is now known that allostery is a fundamental property of all dynamic proteins that exist as an ensemble of conformers with different activities and affinities for substrates. Covalent or non-covalent modifications at allosteric sites shift the equilibrium toward a more active or less active conformer. For comprehensive information on allostery in the cell, see the review in ref. [191]. 

**Figure 6:**
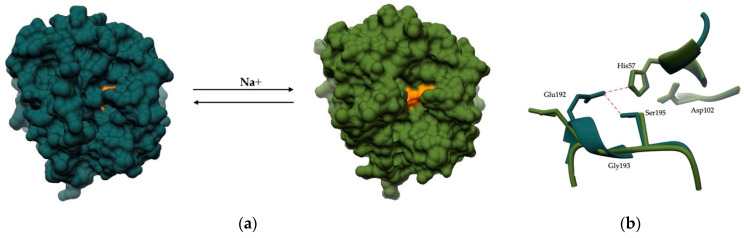
Illustration of the conformational changes of thrombin. (**a**) Protein structures of the “slow” (gray; PDB 1RD3) and “fast” (green; PDB 1PPB) conformations of thrombin. In the “slow” conformation, the closure of the active site can be seen. The transition between conformations is Na^+^ dependent. (**b**) Active site of “slow” and “fast” thrombin. In the “slow” conformation, a rotation of residues Glu192 and Gly193 occurs, allowing the formation of hydrogen bonds (magenta) that undermine the structure of the oxyanion hole.

The main advantage of allosteric over orthosteric inhibition (i.e., binding of the inhibitor to the active site in a substrate-like manner) is the ability to fine-tune enzyme activity. Allosteric inhibitors can act as partial inhibitors of peptidase activity by causing a conformational change in the active site that still allows binding of the substrate but with reduced affinity. One of the best-studied human allosteric peptidases is thrombin, which can act as both a procoagulant and an anticoagulant. The procoagulant function of thrombin involves the conversion of fibrinogen to fibrin and an enhancement of its own activity through a positive feedback loop, whereas the anticoagulant function involves the activation of protein C in the presence of thrombomodulin [192]. Thus, the function of thrombin is dependent on its conformation. The enzyme exists in a “fast” and a “slow” conformation, and the transition between these two is dependent on Na^+^ [193] (Figure 6a). The procoagulant “fast” conformation cleaves fibrinogen with higher specificity and is mainly populated in the presence of Na^+^ ions, whereas the anticoagulant “slow” conformation, which is more abundant in the absence of Na^+^ ions, preferentially cleaves protein C [194]. This difference in substrate specificity of the two conformations is due to conformational changes [193,195,196] involving residues in the active site and Na^+^ binding site [197] (Figure 6b). Thus, the regulation of thrombin activity is complex and is influenced by allosteric communication between the allosteric Na^+^ binding site, exosites I and II, and the active site.

Allosteric regulation was also observed for other trypsin- and chymotrypsin-like serine peptidases. It was found that proteolytic cleavage of the proregion is not required for zymogen activation. Instead, the binding of an allosteric effector mimicking the activating N-terminal peptide to the allosteric site is sufficient to cause a conformational change from an inactive to an active state [7,198]. The regulation of activation of serine peptidases involved in the blood coagulation cascade is also exploited by pathogens such as streptococci and staphylococci. The former secrete streptokinase, which activates plasminogen allosterically [199], and the latter secrete staphylocoagulase, which activates prothrombin in the same manner [200,201]. 

Allosteric regulation has also been extensively studied in human papain-like cysteine peptidases, particularly in cathepsin K. The first known allosteric effectors of this enzyme were GAGs, but since then, many other effectors have been discovered that bind to different sites of the protein and have different effects on its activity [202,203]. Interestingly, in the case of cathepsin K, minimal conformational changes occur during allosteric regulation, as shown by molecular dynamics simulations [204]. However, these changes are reflected in modest changes in activity measured with synthetic substrates. 

**Figure 7:**
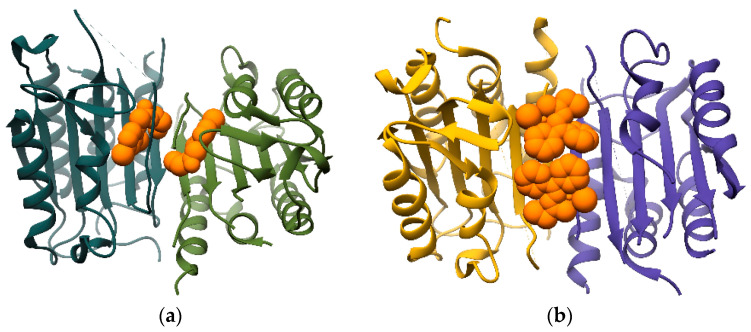
Crystal structures of human caspase-7 in complex with the allosteric inhibitors (**a**) DICA (PDB 1SHJ) and (**b**) NSC321205 (PDB 4FEA). Caspase-7 is shown in cartoon representation and the inhibitors are shown as spheres (orange). Each subunit of the dimer is colored in a different shade.

The activity of caspases can be regulated allosterically at multiple stages. The activation of procaspases can be modulated by allosteric inhibitors that bind to the dimerization site and prevent the formation of an active homodimer. In the case of caspase-9, activation is inhibited in this manner by binding of the BIR3 domain of the X-chromosome linked inhibitor of apoptosis (XIAP) to the dimerization site, keeping the peptidase in an inactive conformation [205]. There are also multiple classes of allosteric caspase inhibitors that act at the level of active enzyme, such as FICA, DICA, designed ankyrin repeat proteins (DARPINs) and a class of copper-containing organometallic compounds [206,207,208,209,210]. A common denominator of these inhibitors is their binding in a 2:2 caspase:inhibitor stoichiometry. The small molecule inhibitor DICA and related compounds bind covalently to a conserved Cys residue near the dimer interface (Figure 7a), whereas the organometallic compound NSC321205 binds non-covalently and directly at the dimer interface (Figure 7b).

In addition to the low specificity of orthosteric inhibitors, the advantage of allosteric inhibitors is that they allow fine-tuning of the different enzyme activities of a single target. An example is gamma-secretase, which cleaves multiple substrates, including β-amyloid precursor protein (APP) and Notch1. Cleavage of these substrates has been linked to the development of Alzheimer’s disease (AD) and cancer, respectively, making gamma-secretase an attractive drug target. The inhibition of total enzyme activity proved insufficient for the treatment of AD because toxicity was caused by the simultaneous inhibition of the Notch pathway [211]. Therefore, researchers have focused on developing allosteric effectors that selectively affect substrate affinity [212,213,214,215]. Beta-secretase or BACE1 is also a potential target for the treatment of AD. However, because of the similarity between cathepsin D, gamma-secretase, and BACE1, orthosteric inhibition could lead to off-target toxicity. Therefore, researchers have developed allosteric inhibitors in the form of small molecules, peptides, and antibodies using various computational and experimental methods [216,217,218,219].

In the case of metalloproteases, mainly computational studies have been performed indicating the presence of allosteric sites [220,221,222]. A rare example of demonstrated allostery in metalloproteases is the angiotensin-converting enzyme, which plays a crucial role in blood pressure regulation by hydrolyzing angiotensin I to angiotensin II. In addition, the enzyme has its own domain that cleaves bradykinin. Because these domains have different substrate preferences, allosteric regulation could allow the specific inhibition of only one of the functions, providing a potential avenue for the regulation of blood pressure and fibrosis [223]. 

Due to increasing bacterial resistance to antibiotics, there is a need for new antibacterial drugs, and bacterial peptidases offer a promising new target. Potential targets include members of the ClpP family, which have been shown to be allosteric (reviewed in reference [224]). These peptidases are responsible for the degradation of defective and misfolded proteins. The ClpP peptidase from *Escherichia coli* has more than 50 different substrates within the cell and is therefore critical for numerous cellular processes, including the regulation of transcription [225]. Activation of these peptidases would lead to uncontrolled protein degradation, ultimately resulting in cell death of the bacterium. Since members of the same family are also found in human mitochondria, the specificity of allosteric effectors is critical. Allosteric inhibitors may also serve as potential antiviral drugs. For example, the major cysteine peptidase of the SARS-CoV-2 virus [82] has been successfully inhibited allosterically. Most synthetically produced inhibitors bind to the dimerization region and prevent the formation of an active homodimer [226,227,228].

## 6. Other Examples of Peptidase Regulation Outside of the Active Site

In the previous sections, the examples presented were described primarily from the point of view of the structural basis for their action. However, there are other examples of modifiers that have not been characterized at the structural level and whose kinetic mode of action differs from the specific inhibition associated with orthosteric inhibitors, which is a strong indication that they do not bind to the active site in a substrate-like manner. For example, mixed and catalytic inhibition mechanisms have been diagnosed for the inhibition of cathepsin B by nitroxoline and its derivatives [229,230]. Indeed, nitroxoline has been shown to bind to the S’ sites of the active site of cathepsin B [229]. Similar kinetic mechanisms were discovered for the inhibition of cathepsin B by caffeic acid and chlorogenic acid [231]. However, these two groups responded differently to changes in pH, suggesting that their mode of action is not identical [231]. 

With complete inhibition, peptidase action is completely suppressed, which is not optimal for cellular functions. However, with partial inhibition, we can maintain the basic activity required for homeostasis. Partial (hyperbolic) inhibition mechanisms were described for allosteric effectors of cathepsin K NSC1334 and NSC94914 [202,203] as well as putative such modifiers of cathepsin K and cathepsin S [203,232]. Several of them, including NSC13345 and NSC94914, showed peculiar kinetic profiles of inhibition and/or activation, depending on the concentration of the substrate [202,203], and altogether, a diverse spectrum of effects ranging from partial specific to partial catalytic inhibition have been observed [203]. Similarly, sulfated coumarins, allosteric inhibitors of thrombin, have been shown to act as partial inhibitors of this peptidase [233]. Partial inhibition is not limited to small molecule modifiers. Various metal ions can partially inhibit not only the activity of cathepsin K but also that of cathepsin S [234]. At the other end of the spectrum, it can also be obtained with proteinaceous binding partners of peptidases that bind away from the active site. Good examples of such action are DARPINs that bind to and inhibit caspase-2 [207] and cathepsin B [235], respectively. The former case is particularly interesting as only 4% of catalytic activity of the peptidase are retained in the complex, and careful kinetic analysis was necessary to discriminate its mode of action from linear inhibition [207]. 

## 7. Non-Inhibitory Binding Partners of Peptidases

A wholly distinct type of regulation of peptidase activity, which is also noteworthy in the context of this review, is the interactions of peptidases with non-inhibitory binding partners. These are typically proteins that bind the peptidase outside its active site without having any appreciable direct effect on the kinetic parameters of enzyme activity. Nevertheless, their presence may influence other aspects of the enzymatic activity of the peptidase, such as stability, localization, etc. One of the best-known categories of such binding partners is membrane proteins, which act as specific receptors for secreted peptidases. A large repertoire of such receptors is available for MMPs, including integrins, tetraspanins, emmprin, etc. (reviewed by Yamamoto et al. [236]). These interactions have been identified for both soluble MMPs [237,238] and membrane-type MMPs, most notably MT-MMP1 [239]. They have been shown to regulate both pericellular proteolysis [237,239] as well as the trafficking and recycling of these peptidases [240]. Well-characterized receptors are also known for the serine peptidase plasmin(ogen) and its activator urokinase. Together, the plasminogen and urokinase plasminogen activator receptors (uPARs) contribute to the activation of plasmin at the cell surface and the concomitant proteolytic destruction of the pericellular extracellular matrix that is the hallmark of many cancers [241]. Not surprisingly, these interactions are considered potential targets for anticancer therapies [242] as well as diagnostic imaging [243]. Similarly, cysteine cathepsin B, an otherwise unstable peptidase in the extracellular environment [244] which is also associated with cancer progression [245], was found to mediate pericellular proteolysis by binding to caveolin-1 [246,247] and annexin II [248]. Unfortunately, this type of interaction has remained uninvestigated for most peptidases and thus potentially representing a significant untapped potential for drug targeting that is worth exploring in the future.

Alternatively, interactions with non-inhibitory binding partners may mediate non-enzymatic physiological and/or pathological functions of peptidases. A well-known example is the transmembrane form of dipeptidyl-peptidase 4 (DPP4), an important target in type 2 diabetes, which not only regulates immune cells via its enzymatic activity but also functions as a cell surface receptor (reviewed in [249]). For example, its interactions with extracellular adenosine deaminase [250] and CD45 [251] regulate the activation of T cells. In addition, DPP4 has been identified as the entry receptor for Middle East respiratory syndrome (MERS) coronavirus (CoV) [252]. Another much more infamous example of peptidases as viral entry points is the cell surface metalloprotease angiotensin-converting enzyme 2 (ACE2), which serves as the receptor used by SARS-CoV [253,254]. Disruption of this interaction by neutralizing antibodies has been identified as one of the key elements for a successful immune response to infection [255]. It is therefore not surprising that ACE2 mimetics, which act as scavengers of viral particles, are emerging as treatment for SARS-CoV infections [256]. Non-proteolytic functions of peptidases may also contribute to the pathogenicity of various microbial pathogens. In this case, the culprits are microbial peptidases that contribute to host invasion (reviewed by Jarocki et al. [257]). Examples include streptopain (SpeB) and C5a from streptococci, which mediate the adhesion of bacterial cells to structural proteins of the extracellular matrix [258,259], plasminogen activator from *Yersinia pestis*, etc. Such interactions, too, are potentially druggable and may provide novel opportunities for disease management.

## 8. Conclusions

Many peptidases have been implicated in various human diseases characterized by either up- or down-regulation of their catalytic activity. Given their involvement in pathological processes, the study of the regulation of proteolytic activity has attracted considerable interest. Moreover, proteolytic enzymes of pathogens are potential targets for new generations of antibiotics. Looking at the development of drugs in clinical trials, it is reasonable to assume that most of the drugs currently being tested will unfortunately not reach clinical application [260]. The problem usually lies in the specificity of the drugs, which not only bind to the target protein but also have unexpected interactions with other peptidases [261]. To overcome this challenge, recent research has focused on finding novel ways to regulate peptidase activity, such as by regulating zymogen activation, modulating enzyme activity via the binding of effectors to exosites, and allosteric sites. These mechanisms offer potential opportunities for targeting human, viral, and bacterial proteolytic enzymes. However, it is critical to note that the regulation of peptidase activity is a complex process that results from the sum of multiple mechanisms, as seen in the case of thrombin.

## Data Availability

Not applicable.
